# Common meat inspection findings in sheep in Finland

**DOI:** 10.1186/s13028-026-00895-0

**Published:** 2026-11-13

**Authors:** Jenni Stenius, Riina Keski-Saari, Nina Nihtilä, Heli Nordgren, Eeva Mustonen, Heli Lindeberg, Johanna Rautiainen, Maria Fredriksson-Ahomaa, Riikka Laukkanen-Ninios

**Affiliations:** 1 Department of Food Hygiene and Environmental Health, Faculty of Veterinary Medicine, University of Helsinki, P.O. Box 66, Helsinki, FI-00014 Finland; 2 Department of Veterinary Biosciences, Faculty of Veterinary Medicine, University of Helsinki, P.O. Box 66, Helsinki, FI-00014 Finland; 3 Department of Production Animal Medicine, Faculty of Veterinary Medicine, University of Helsinki, Paroninkuja 20, Saarentaus, FI-04920 Finland; 4 Natural Resources Institute Finland (Luke), Halolantie 31 A, Maaninka, FI-71750 Finland; 5 Sheep World Ltd, Metsälammentie 20, Ylinen, FI-34130 Finland

**Keywords:** Condemnation, Conventional production, Lamb, Lesion, Meat inspection, Organic production, Prevalence, Sheep

## Abstract

**Background:**

Sheep meat is produced on a small scale in Finland, with ca. 50,000 animals slaughtered annually. In 2024, over 20% of sheep meat produced in Finland was organic. Legislative requirements differ between organic and conventional production, which may be reflected in differences in animal health and welfare between the two production systems. Meat inspection data can be used to assess the health and welfare of production animals. However, studies on meat inspection findings in sheep and comparative research on lesion and condemnation prevalences between the two production systems are scarce. In this study, data were collected from a slaughterhouse that received over 20% (*n* = 11,355) of all sheep slaughtered in Finland in the year 2024. Lambs (sheep under one year of age) constituted 79% of the slaughtered animals, with 37% of all animals being organically reared.

**Results:**

No lesions or condemnations were recorded in 96% of all carcasses originating from 132/228 (58%) farms. Whole- and partial-carcass condemnations were more frequent in adult sheep than in lambs in both organic and conventional production, but no significant difference was observed between the production systems. The lesions with the highest animal and farm prevalences, respectively, were dicrocoeliasis caused by the lancet liver fluke *Dicrocoelium dendriticum* (2.5% and 3.5%), pleuritis (0.8% and 22.4%), bone fracture (0.4% and 9.7%), acute bruise (0.3% and 6.6%), pneumonia (0.2% and 7.0%), and liver abscesses (0.1% and 3.9%). The prevalence of *D. dendriticum* was significantly higher in organic production (*p* < 0.001) and in adults (*p* < 0.001) than lambs. Additionally, dicrocoeliasis was a farm-specific problem, with only eight positive farms (3.5%). In addition to dicrocoeliasis, pleuritis was also age-related, with a significantly higher prevalence in adults than in lambs in both production systems.

**Conclusions:**

The results indicate a good health status of sheep in Finland. Differences between animal age groups seem to be greater than those between production systems. Meat inspection results can help identify underlying health and welfare issues on the farm. However, since the results may be affected by such factors as animal nutrition, more research on lesion etiology in Finland is required.

## Background

Sheep farming in Finland is conducted on a relatively small scale. In 2024, there were approximately 115,400 sheep on a total of 1,112 farms, with the Finnsheep being the most common breed [1, 2​]. In 2024, around 50,000 sheep were slaughtered [2, 3]. For comparison, beef production was more than 82-fold and pork production more than 150-fold that of sheep meat [2]. Moreover, sheep meat is considerably more likely to be produced to organic standards than other types of meat. In 2024, over 20% of sheep meat (by weight) was organic, whereas the corresponding proportions for beef and pork were less than 5% and 0.5% [2].

Meat inspection, i.e. antemortem and postmortem inspection, is performed at the slaughterhouse by an official veterinarian [4] employed by the Finnish Food Authority (FFA). Postmortem inspection can provide information about animal welfare, in addition to the overall health status of the farm and its success in animal husbandry [5]. Therefore, meat inspection data can help identify and control health and welfare issues on the farm [5]. Despite the volume of meat inspection data collected at slaughter, it is not fully utilized [5].

Data on meat inspection findings in sheep are fairly scarce, but sheep are generally deemed to be healthy and the meat classified as fit for human consumption, with only a small number of carcasses condemned or lesions detected at official meat inspection [6–10]. In general, lesions related to lungs and liver have been among the most common lesions recorded in sheep meat inspection [6–10]. In a previous Finnish study, lung lesions were the most common lesions in sheep meat inspection, followed by various types of liver lesions and occasional heart and kidney lesions [6].

Legislation sets additional demands for organic production relative to conventional production, such as better access to pasture and restricted use of medical treatments [11]. These requirements may have implications for animal health and welfare, which could subsequently be reflected in meat inspection data. However, few comparative studies in sheep exist.

Previous studies suggest that differences in animal health and welfare between organic and conventional sheep production systems are generally modest. No clear distinctions were found in a comprehensive multi-species review [12]. Parasites, especially gastrointestinal nematodes and lancet liver flukes (*Dicrocoelium dendriticum*), seem to be more common in organic sheep [7, 12, 13]. However, organic sheep have been reported to exhibit a lower prevalence of non-parasitic liver diseases [7]. Preventive husbandry strategies and management based on early detection appear to be critical components in mitigating health and welfare challenges in both production systems [12, 14].

Animal age may influence lesion prevalence, although this has not been widely investigated in sheep [8, 9]. A previous Finnish study found no association between the prevalence of meat inspection findings and animal age [6]. However, studies conducted in the UK report an age-related increase in pleuritic, pneumonic and arthritic lesions, together with more frequent abscesses and liver fluke findings [8, 9].

Here, we studied carcass condemnations and lesions recorded at meat inspection in organically and conventionally produced sheep. The aim was to determine the most common meat inspection findings in Finland and to compare their animal and farm prevalences in organically and conventionally raised lambs and adult sheep. The results can help farms and slaughterhouses direct their resources more efficiently to improve animal welfare and meat quality and to reduce food loss.

## Materials and methods

The meat inspection and slaughter data for 2024 were obtained from a major sheep slaughterhouse in Finland. Animals with recorded lesions were included in the meat inspection dataset. The data included meat inspection decision codes (partially condemned or totally condemned), a brief written description of the official veterinarian’s observations, and lesion codes based on the meat inspection coding system of the Finnish Food Authority (FFA) [15]. Whole-carcass condemnation referred to condemnation of the carcass, organs, and blood, while partial condemnation primarily referred to the condemnation of varying parts of the carcass, excluding the organs and blood [15]. Pleural adhesions in the carcass were included in partial condemnations. The animal-level data were coded based on meat inspection decisions and lesion codes. Additional information on some of the lesions (bone fractures and bruises) was collected from the written descriptions. According to FFA guidelines, small lesions requiring minor trimming from the carcass do not necessarily need to be recorded as partial condemnations [15].

Slaughter age, farm of origin (pseudonymized), and production type (organic/conventional) of all slaughtered animals were collected from the slaughterhouse’s database in an Excel file. Animals younger than one year were classified as lambs and all others as adults [16, 17].

### Statistical data analysis

Data were managed in Excel (Microsoft Excel for Microsoft 365, MSO version 2502 Build 16.0.18526.20546), and farm-level aggregated data were created using SPSS (IBM SPSS Statistics, version 30.0.0.0). Animal-level prevalences in each group (all animals, organic adults and lambs, conventional adults and lambs) were calculated by dividing the number of cases by the total number of animals in the group. Farm prevalences were similarly calculated by dividing the number of positive farms by the total number of farms in a group. Each farm could belong to multiple groups, e.g. a farm with both organic adults and organic lambs. Confidence limits were calculated using Binomial (Clopper-Pearson exact) method in SPSS. Pairwise comparisons were conducted using Fisher’s exact test in SPSS. *p*-values were adjusted for multiple comparisons using the Holm method, and results with *p* < 0.05 were considered significant. Differences were tested between 1) adult sheep and lambs, 2) organic and conventional production types, 3) organic adults and organic lambs, 4) conventional adults and conventional lambs, 5) organic adults and conventional adults, and 6) organic lambs and conventional lambs. Figures were created using Excel.

### Description of the farms in the dataset

The data comprised 11,355 animals that originated from 228 farms (Table 1). Of these farms, 175 supplied only conventionally produced animals, 44 only organic animals, and 9 both categories. Both adults and lambs were slaughtered from 142 farms, only adults from 31 farms, and only lambs from 55 farms (Table 1).

**Table 1:** Description of farms and sheep in the study

Production type / age group		N of farms	N of slaughtered animals	N of slaughtered animals per farm			
				Min	Median	Mean	Max
Total		228	11,355	1	29	50	488
Adults		173	2,433	1	9	14	84
Lambs		197	8,922	1	28	45	419
Conventional		184	7,177	1	20	39	488
Adults		132	1,552	1	7	12	80
Lambs		155	5,625	1	17	36	419
Organic		53	4,178	11	62	79	437
Adults		46	881	1	16	19	63
Lambs		50	3,297	2	54	66	387

The data used to assess meat inspection findings in sheep in Finland comprised 11,355 slaughtered animals that originated from 228 farms. Each farm could belong to multiple groups, e.g. a farm with both organic adults and organic lambs. Animals older than one year were classified as adults and younger ones as lambs. *N*: number

## Results

Overall, 10,867 animals (96%) originating from 136 farms (60%) presented no detectable lesions, and therefore, no condemnations at meat inspection were recorded.

### Condemnations

Of all carcasses, 0.1% were totally condemned and 1.7% partially condemned (Table 2). Whole-carcass condemnations originated from 11 farms (4.8%), and partial condemnations from 75 farms (32.9%) (Table 3).

**Table 2:** Animal-level prevalence of carcass condemnations in sheep in Finland

Production type / age group		N of slaughtered animals	Whole-carcass condemnation			Partial-carcass condemnation		
			n	%	95% CI	n	%	95% CI
Total		11,355	16	0.14	0.08–0.23	189	1.66	1.44–1.92
Adults		2,433	15	0.62	0.35–1.01	74	3.04	2.40–3.80
Lambs		8,922	1	0.01	0.00–0.06	115	1.29	1.07–1.55
Conventional		7,177	9	0.13	0.06–0.24	121	1.69	1.40–2.01
Adults		1,552	8	0.52	0.22–1.01	46	2.96	2.18–3.94
Lambs		5,625	1	0.02	0.00–0.10	75	1.33	1.05–1.67
Organic		4,178	7	0.17	0.07–0.34	68	1.63	1.27–2.06
Adults		881	7	0.79	0.32–1.63	28	3.18	2.12–4.56
Lambs		3,297	0	0	0.00–0.11	40	1.21	0.87–1.65

**N:**number of animals in the group;*n*: number of positive animals;%: prevalence as percentage;*95% CI*: 95% confidence interval. Animals older than one year were classified as adults and younger ones as lambs

**Table 3:** Farm prevalence of carcass condemnations in sheep in Finland

Animal group		N of farms	Whole-carcass condemnation			Partial-carcass condemnation		
			n	%	95% CI	n	%	95% CI
Total		228	11	4.82	2.43–8.47	75	32.89	26.84–39.41
Adults		173	10	5.78	2.81–10.37	35	20.23	14.51–27.00
Lambs		197	1	0.51	0.01–2.80	56	28.43	22.24–35.27
Conventional		184	6	3.26	1.21–6.96	51	27.71	21.39–34.78
Adults		132	5	3.79	1.24–8.62	22	16.67	10.75–24.14
Lambs		155	1	0.65	0.02–3.54	38	24.52	17.97–32.06
Organic		53	5	9.43	3.13–20.66	24	45.28	31.56–59.55
Adults		46	5	10.87	3.62–23.57	13	28.26	15.99–43.46
Lambs		50	0	0.00	0.00–7.11	18	36.00	22.92–50.81

N:number of farms in the group; n: number of positive farms; %: prevalence as percentage; 95% CI: 95% confidence interval. Animals older than one year were classified as adults and younger ones as lambs

**Fig. 1:**
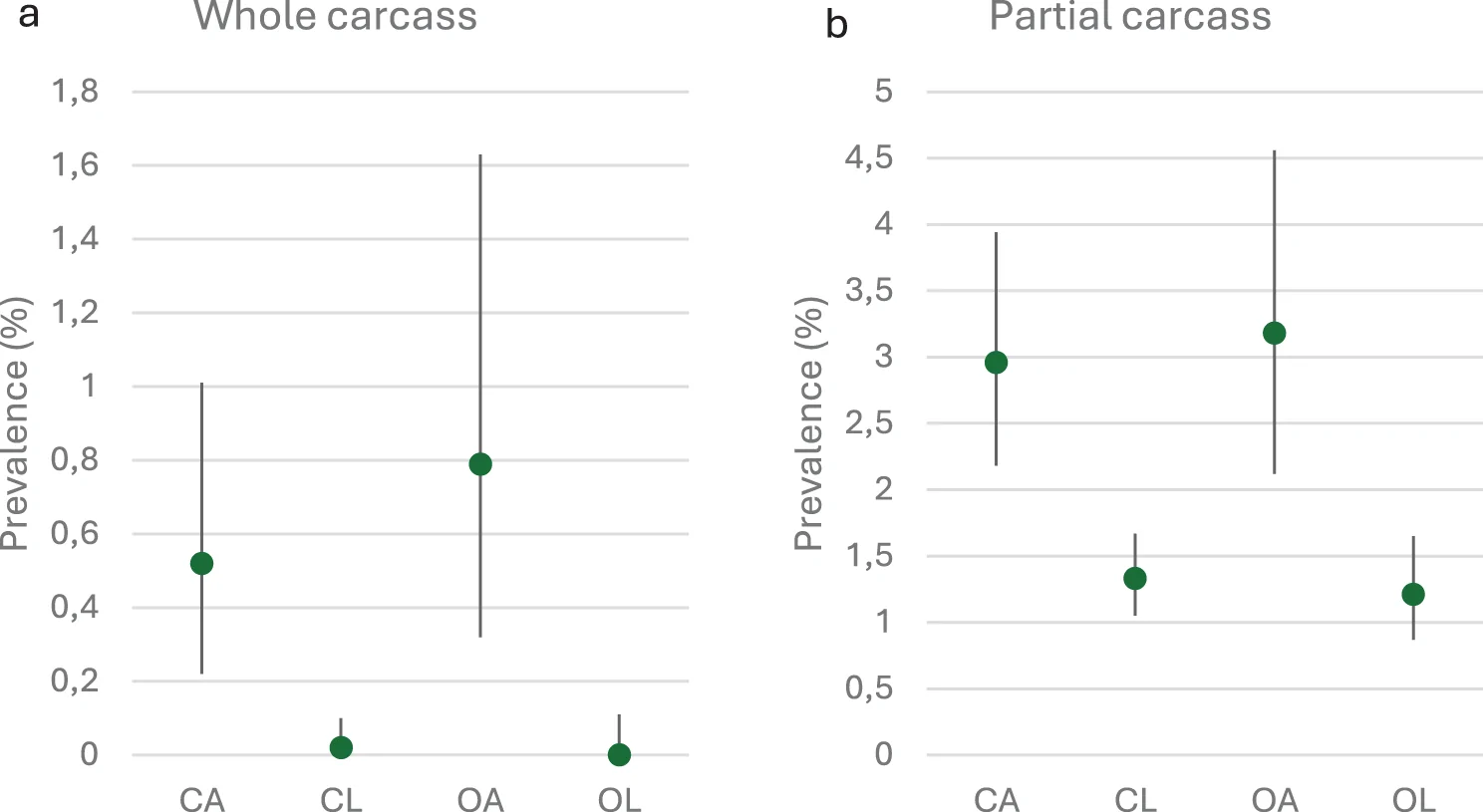
Animal prevalences and confidence intervals of carcass condemnations. *N*: number of animals in the group; *CA*: conventional adult; *CL*: conventional lamb; *OA*: organic adult; *OL*: organic lamb. Animals older than one year were classified as adults and younger ones as lambs. Prevalence is marked by a green spot, lines above and under the spot represent 95% confidence intervals

The prevalence of whole-carcass condemnations (*p* < 0.001) was significantly higher in adults than in lambs when production type was not considered but also in both organic (*p* = 0.002) and conventional (*p* = 0.005) productions when production type was considered (Tables 2 and 4, Fig. 1). Similarly, the prevalence of partial condemnations (*p* < 0.001) was significantly higher in adults than in lambs when production type was not considered but also in both organic (*p* = 0.018) and conventional (*p* = 0.006) productions when production type was considered.

**Table 4:** Significant differences between tested groups of condemnations and meat inspection lesions

Comparison	Lesion	Holm corrected *p*-value
Adult sheep vs. lambs	Total condemnation	<0.0001
	Partial condemnation	<0.0001
	Dicrocoeliasis	<0.0001
	Pleuritis	<0.0001
Organic vs. conventional production	Dicrocoeliasis	0.0001
Organic adults vs. organic lambs	Total condemnation	0.0025
	Partial condemnation	0.0183
	Dicrocoeliasis	<0.0001
	Pleuritis	0.0012
Conventional adults vs. conventional lambs	Total condemnation	0.0046
	Partial condemnation	0.0061
	Dicrocoeliasis	<0.0001
	Pleuritis	0.0151
Organic adults vs. conventional adults	Dicrocoeliasis	0.0041

*p*-value: significance, tested using Fisher’s exact test with Holm‑adjusted P‑values. Animals older than one year were classified as adults and younger ones as lambs

No significant differences (*p* > 0.05) emerged in the prevalence of whole- or partial-carcass condemnation between organically and conventionally produced animals when the age group was not considered or when it was considered (Tables 2 and 4).

**Fig. 2:**
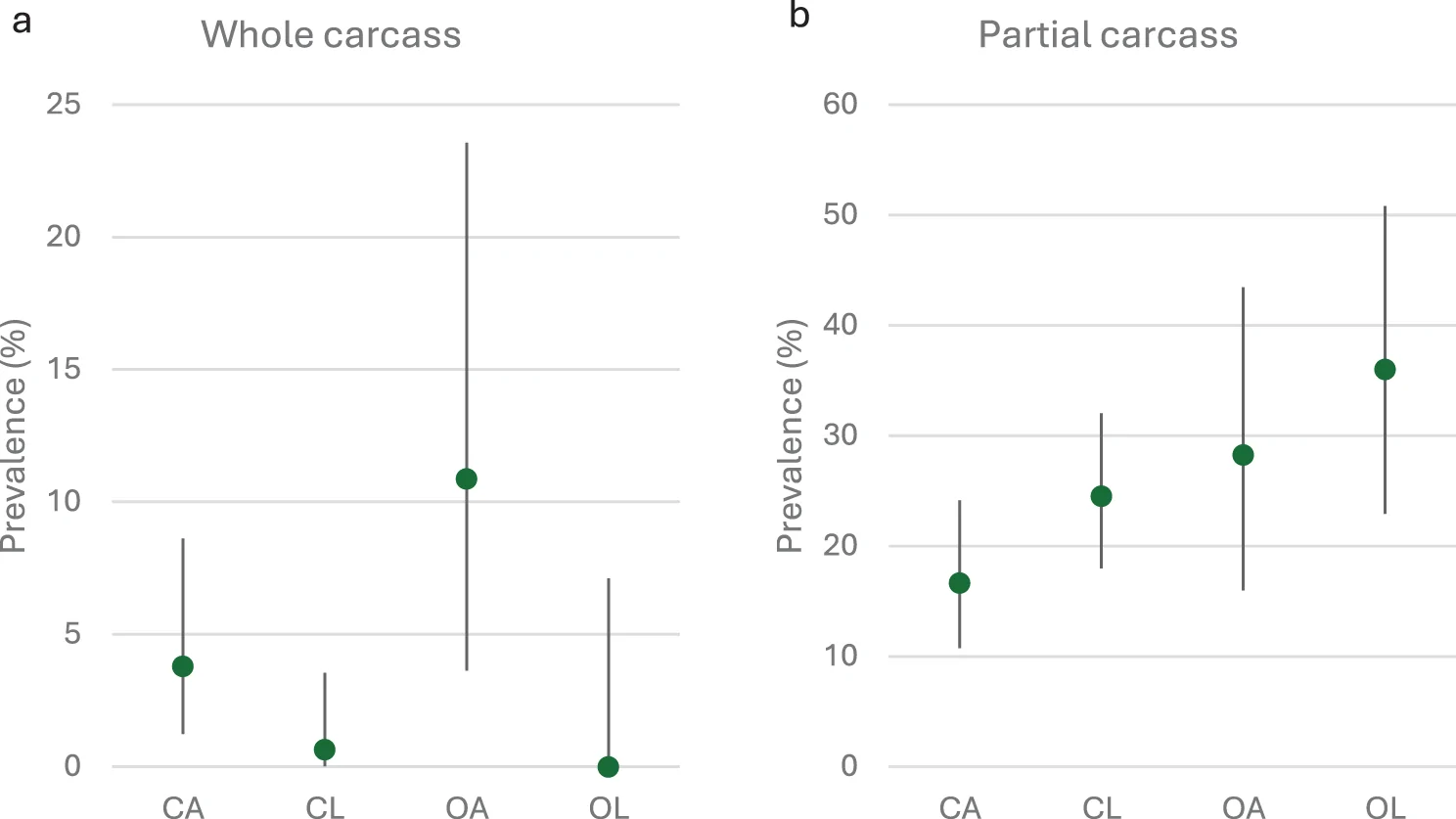
Farm prevalences and their confidence intervals of carcass condemnations. *N*: number of farms in the group; *CA*: conventional adult; *CL*: conventional lamb; *OA*: organic adult; *OL*: organic lamb. Animals older than one year were classified as adults and younger ones as lambs. Prevalence is marked by a green spot, lines above and under the spot represent 95% confidence intervals

There were no significant differences in farm prevalences of total or partial condemnations (Tables 3 and 4, Fig. 2).

### Lesions

In total, 20 different types of meat inspection lesions were recorded (Tables 5 and 6). At least one lesion was recorded in 488 carcasses (4.3%), originating from 92 farms (40.4%). This article will focus on the six most common lesions, which had a prevalence of at least 0.1% (over 10 animals): dicrocoeliasis (2.5%), pleuritis (0.8%), bone fracture (0.4%), acute bruise (0.3%), pneumonia (0.2%), and liver abscesses (0.1%) (Table 5).

**Table 5:** Prevalence of meat inspection findings in animals in sheep in Finland

Lesion	Total (*N* = 11,355)				Conventional							Organic						
					Adult (*N* = 1,552)				Lamb (*N* = 5,625)			Adult (*N* = 881)				Lamb (*N* = 3,297)		
	Pos	%	95% CI		Pos	%	95% CI		Pos	%	95% CI	Pos	%	95% CI		Pos	%	95% CI
Dicrocoeliasis	285	2.51	2.23–2.81		60	3.87	3.02–5.05		80	1.42	1.13–1.77	70	7.95	6.25–9.93		75	2.27	1.79–2.84
Pleuritis	86	0.76	0.61–0.93		24	1.55	1.01–2.34		28	0.50	0.33–0.72	19	2.16	1.30–3.35		15	0.45	0.25–0.75
Bone fracture	41	0.36	0.26–0.49		7	0.45	0.19–0.95		22	0.39	0.25–0.59	4	0.45	0.12–1.16		8	0.24	0.10–0.48
Acute bruise	36	0.32	0.22–0.44		0	0.00	0.00–0.24		21	0.37	0.23–0.57	1	0.11	0.00–0.63		14	0.42	0.23–0.71
Pneumonia	20	0.18	0.11–0.27		5	0.32	0.11–0.76		7	0.12	0.05–0.26	5	0.57	0.18–1.32		3	0.09	0.02–0.27
Liver abscesses	11	0.10	0.05–0.17		1	0.06	0.00–0.37		1	0.02	0.00–0.10	3	0.34	0.07–0.99		6	0.18	0.07–0.40
Kidney lesions	9	0.08	0.04–0.15		0	0.00	0.00–0.24		4	0.07	0.02–0.18	1	0.11	0.00–0.63		4	0.12	0.03–0.31
Generalized infection/sepsis	5	0.04	0.01–0.08		3	0.19	0.04–0.57		0	0.00	0.00–0.07	2	0.23	0.03–0.82		0	0.00	0.00–0.11
Abscess(es)	4	0.04	0.01–0.09		1	0.06	0.00–0.37		1	0.02	0.00–0.10	1	0.11	0.00–0.63		1	0.03	0.00–0.17
Pericarditis	4	0.04	0.01–0.09		1	0.06	0.00–0.37		1	0.02	0.00–0.10	2	0.23	0.03–0.82		0	0.00	0.00–0.11
Emaciated, cachexia	3	0.03	0.01–0.08		1	0.06	0.00–0.37		1	0.02	0.00–0.10	1	0.11	0.00–0.63		0	0.00	0.00–0.11
Fatty liver	3	0.03	0.01–0.08		1	0.06	0.00–0.37		2	0.04	0.00–0.13	0	0.00	0.00–0.42		0	0.00	0.00–0.11
Liver cirrhosis	3	0.03	0.01–0.08		1	0.06	0.00–0.37		2	0.04	0.00–0.13	0	0.00	0.00–0.42		0	0.00	0.00–0.11
Abnormal carcass color	2	0.02	0.00–0.06		2	0.13	0.02–0.47		0	0.00	0.00–0.07	0	0.00	0.00–0.42		0	0.00	0.00–0.11
Icterus	2	0.02	0.00–0.06		1	0.06	0.00–0.37		0	0.00	0.00–0.07	1	0.11	0.00–0.63		0	0.00	0.00–0.11
Heart condition	1	0.01	0.00–0.05		0	0.00	0.00–0.24		0	0.00	0.00–0.07	0	0.00	0.00–0.42		1	0.03	0.00–0.17
Serositis	1	0.01	0.00–0.05		0	0.00	0.00–0.24		0	0.00	0.00–0.07	0	0.00	0.00–0.42		1	0.03	0.00–0.17
Other lesion, liver	1	0.01	0.00–0.05		1	0.06	0.00–0.37		0	0.00	0.00–0.07	0	0.00	0.00–0.42		0	0.00	0.00–0.11
Chronic bruise	1	0.01	0.00–0.05		0	0.00	0.00–0.24		1	0.02	0.00–0.10	0	0.00	0.00–0.42		0	0.00	0.00–0.11
Tumor(s)	1	0.01	0.00–0.05		1	0.06	0.00–0.37		0	0.00	0.00–0.07	0	0.00	0.00–00.42		0	0.00	0.00–0.11

N:number of animals in the group; Pos: number of positive animals; %: prevalence as percentage; 95% CI: 95% confidence interval. Animals older than one year were classified as adults and younger ones as lambs

**Table 6:** Farm prevalence of lesions found in meat inspection in sheep in Finland

Lesion	Total (*N* = 228)				Conventional							Organic						
					Adult (*N* = 132)				Lamb (*N* = 155)			Adult (*N* = 46)				Lamb (*N* = 50)		
	Pos	%	95% CI		Pos	%	95% CI		Pos	%	95% CI	Pos	%	95% CI		Pos	%	95% CI
Pleuritis	51	22.37	17.13–20.34		16	12.12	7.09–18.94		19	12.26	7.54–18.48	13	28.26	15.99–43.46		9	18.00	8.58–31.44
Bone fracture	22	9.65	6.15–14.24		4	3.03	0.83–7.58		12	7.74	4.06–13.13	3	6.52	1.37–17.90		5	10.00	3.33–21.81
Pneumonia	16	7.02	4.06–11.15		5	3.79	1.24–8.62		6	3.87	1.43–8.23	4	8.70	2.42–20.79		2	4.00	0.49–13.71
Acute bruise	15	6.58	3.73–10.62		0	0.00	0.00–2.76		9	5.81	2.69–10.74	1	2.17	0.06–11.53		5	10.00	3.33–21.81
Liver abscesses	9	3.95	1.82–7.36		1	0.76	0.02–4.15		1	0.65	0.02–3.54	2	4.35	0.53–14.84		5	10.00	3.33–21.81
Dicrocoeliasis	8	3.51	1.53–6.80		6	4.55	1.69–9.63		2	1.29	0.16–4.58	2	4.35	0.53–14.84		2	4.00	0.49–13.71
Kidney lesions	8	3.51	1.53–6.80		0	0.00	0.00–2.76		3	1.94	0.40–5.55	1	2.17	0.06–11.53		4	8.00	2.22–19.23
Generalized infection	5	2.19	0.72–5.04		3	2.27	0.47–6.50		0	0.00	0.00–2.35	2	4.35	0.53–14.84		0	0.00	0.00–7.11
Abscess(es)	4	1.75	0.48–4.43		1	0.76	0.02–4.15		1	0.65	0.02–3.54	1	2.17	0.06–11.53		1	2.00	0.05–10.65
Pericarditis	4	1.75	0.48–4.43		1	0.76	0.02–4.15		1	0.65	0.02–3.54	2	4.35	0.53–14.84		0	0.00	0.00–7.11
Emaciated	3	1.32	0.27–3.80		1	0.76	0.02–4.15		1	0.65	0.02–3.54	1	2.17	0.06–11.53		0	0.00	0.00–7.11
Fatty liver	3	1.32	0.27–3.80		1	0.76	0.02–4.15		2	1.29	0.16–4.58	0	0.00	0.00–7.71		0	0.00	0.00–7.11
Abnormal color	2	0.88	0.11–3.13		2	1.52	0.18–5.37		0	0.00	0.00–2.35	0	0.00	0.00–7.71		0	0.00	0.00–7.11
Icterus	2	0.88	0.11–3.13		1	0.76	0.02–4.15		0	0.00	0.00–2.35	1	2.17	0.06–11.53		0	0.00	0.00–7.11
Liver cirrhosis	2	0.88	0.11–3.13		1	0.76	0.02–4.15		1	0.65	0.02–3.54	0	0.00	0.00–7.71		0	0.00	0.00–7.11
Chronic bruise	1	0.44	0,01–2,42		0	0.00	0.00–2.76		1	0.65	0.02–3.54	0	0.00	0.00–7.71		0	0.00	0.00–7.11
Heart condition	1	0.44	0.01–2.42		0	0.00	0.00–2.76		0	0.00	0.00–2.35	0	0.00	0.00–7.71		1	2.00	0.05–10.65
Tumor(s)	1	0.44	0.01–2.42		1	0.76	0.02–4.15		0	0.00	0.00–2.35	0	0.00	0.00–7.71		0	0.00	0.00–7.11
Other lesion, liver	1	0.44	0.01–2.42		1	0.76	0.02–4.15		0	0.00	0.00–2.35	0	0.00	0.00–7.71		0	0.00	0.00–7.11
Serositis	1	0.44	0.01–2.42		0	0.00	0.00–2.76		0	0.00	0.00–2.35	0	0.00	0.00–7.71		1	2.00	0.05–10.65

N:number of farms in the group; Pos: number of positive farms; %: prevalence as percentage; 95% CI: 95% confidence interval. Animals older than one year were classified as adults and younger ones as lambs

**Fig. 3:**
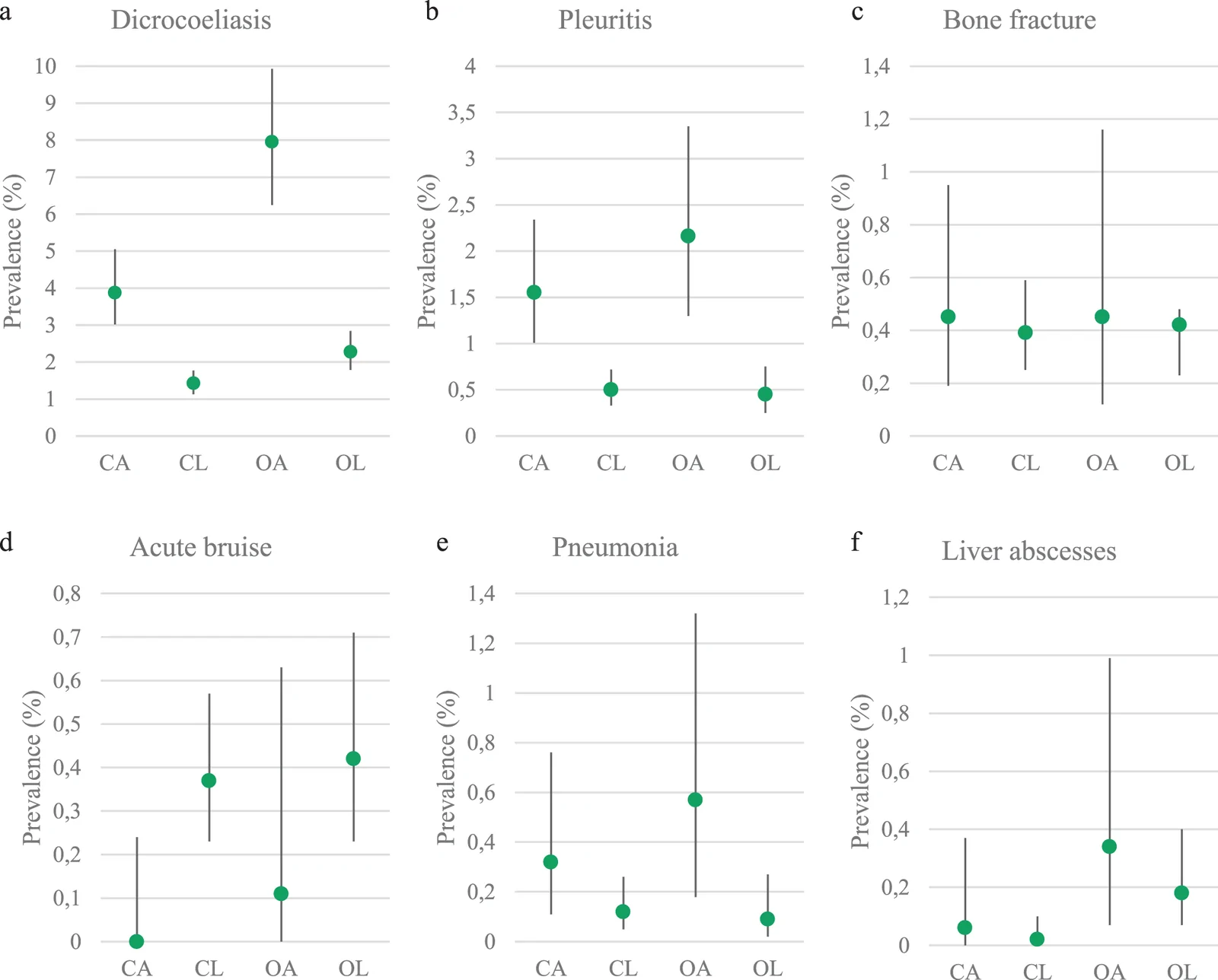
Animal prevalences and their confidence intervals of the six most common lesions. *N*: number of animals in the group; *CA*: conventional adult; *CL*: conventional lamb; *OA*: organic adult; *OL*: organic lamb. Animals older than one year were classified as adults and younger ones as lambs. Prevalence is marked by a green spot, lines above and under the spot represent 95% confidence intervals

The prevalence of dicrocoeliasis was higher in adults than in lambs for both production types (P* < *0.001) (Table 4). In addition, the prevalence was higher in organic production than in conventional production (*p* < 0.001) and in organic adults than in conventional adults (*p* = 0.004). The prevalence of dicrocoeliasis was not significantly higher in organic lambs (P*>*0.05) than in conventional lambs (Table 5 and Fig. 3).

The prevalence of pleuritis was higher in adults than in lambs (*p* < 0.001) when production type was not considered and also when it was considered (organic, *p* = 0.001; conventional, *p* = 0.015).

No other significant differences (P*>*0.05) in lesion prevalences were detected between production types or animal age groups at the animal level (Table 4).

**Fig. 4:**
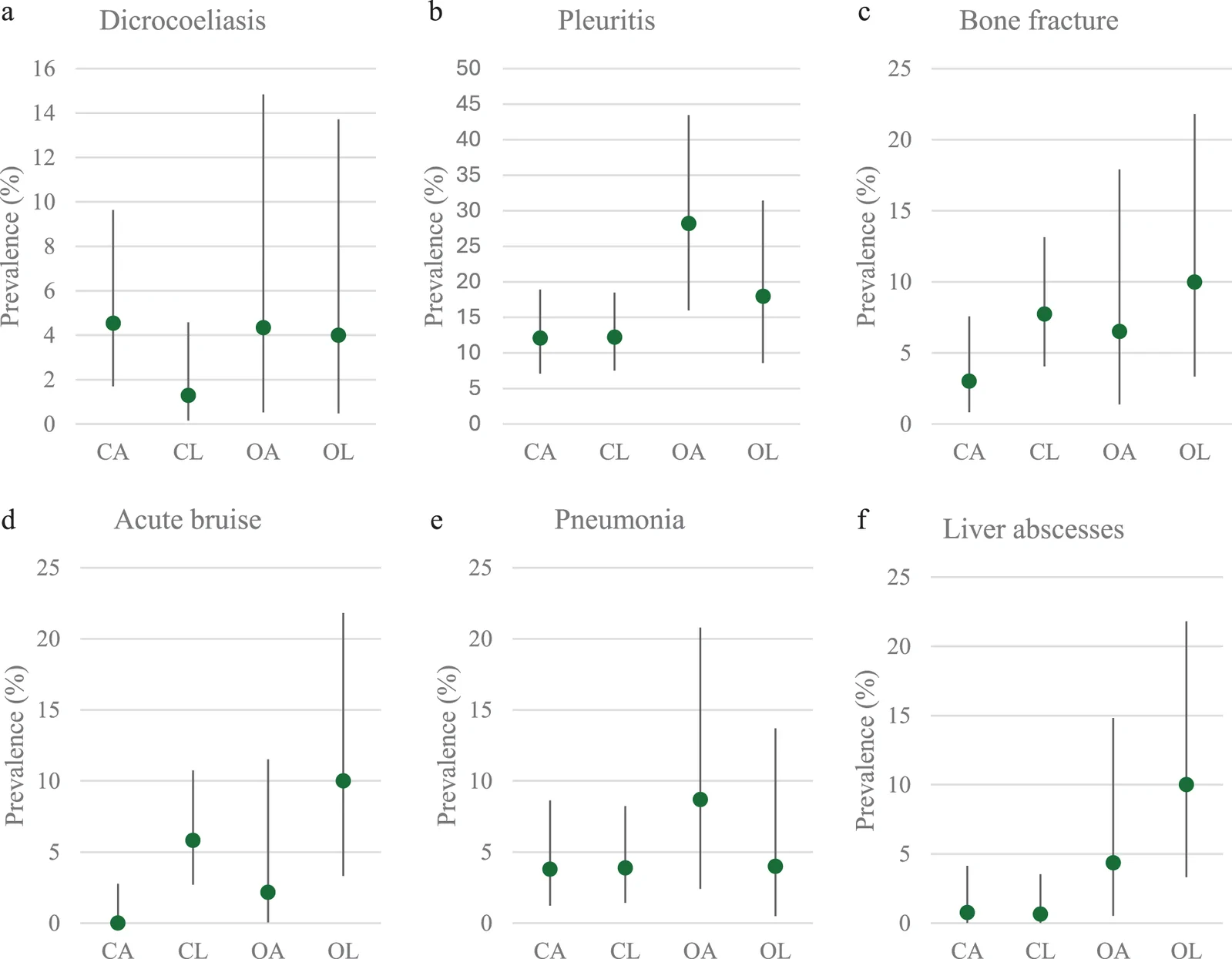
Farm prevalences and their confidence intervals of the six most common lesions. *N*: number of farms in the group; *CA*: conventional adult; *CL*: conventional lamb; *OA*: organic adult; *OL*: organic lamb. Animals older than one year were classified as adults and younger ones as lambs. Prevalence is marked by a green spot, lines above and under the spot represent 95% confidence intervals

The highest farm prevalences were pleuritis (22.4%), bone fracture (9.6%), pneumonia (7.0%), acute bruise (6.6%), liver abscesses (3.9%), and dicrocoeliasis (3.5%) (Table 6, Fig. 4). No significant differences (P*>*0.05) were observed between production types or animal age groups.

Based on written descriptions in the meat inspection data, rib fractures accounted for 97.6% (40/41) of all bone fractures, and among these cases, 45.0% of the animals (18/40) had fractures in two ribs or more. Of the rib fractures, 73.1% occurred in lambs (30/41), with a prevalence of 0.3% (29/8,922) in lambs and 0.5% (11/2,433) in adults and 0.3% (12/4,178) in organic production and 0.4% (28/7,166) in conventional production. Animals with rib fractures originated from 21/228 farms (9.2%), with a farm prevalence of 13.2% (7/53) in organic production and 7.6% (14/184) in conventional production. Additionally, 83.3% (30/36) of all acute bruises were described as ecchymoses in the abdominal wall. These ecchymoses occurred only in lambs. The prevalence of ecchymoses was 0.4% (12/3,298) in organic lambs and 0.3% (18/5,625) in conventional lambs. The lambs with ecchymoses originated from 10/197 farms (5.1%), with a prevalence of 8.0% (4/50) in organic and 3.9% (6/155) in conventional production.

## Discussion

### Condemnations

The prevalence of whole-carcass condemnations in sheep (0.1%) and on farm level (4.8%) was low, indicating good animal health. The prevalence in sheep is in line with previous European studies, in which it has varied between 0.1% and 0.6% [18–21]. In our study, the prevalence of both whole- and partial-carcass condemnations was significantly higher in adults than in lambs. Older sheep have had more time to develop disease and may have been removed from the herd for health- or age-related reasons [8, 9]. The prevalence of whole- and partial-carcass condemnations did not vary between conventional and organic production systems, suggesting that organic and conventional farms produce animals of similar health status. We found no previous studies comparing condemnations between organic and conventional production types or young and old animals.

### Common meat inspection lesions

The most common meat inspection lesions in our study were dicrocoeliasis, pleuritis, fractures, acute bruises, pneumonia, and liver abscesses. Similar results have been reported elsewhere in Europe; dicrocoeliasis, pleuritis and pneumonia in Sweden, and pleuritis, pneumonia, liver abscesses and bruises in the UK [7–9]. However, in some countries, such as the UK and Poland, fascioliasis (liver fluke) instead of dicrocoeliasis was detected in sheep during meat inspection [8, 9, 21], suggesting differences in the geographical distribution of different liver flukes. In our study, lungworms were not recorded as a separate finding, but were detected and recorded during meat inspection in Sweden [7] and the UK [8, 9]. No published data were found on lungworms in sheep in Finland. However, *Müllerius (Muellerius) capillaris* and *Protostrongylus rufescens* have been recorded in Finland, unlike *Dictyocaulus filaria* [22].

Published data on meat inspection lesions in different countries are scarce. In addition, no country comparisons of how meat inspection data are recorded and collected from sheep slaughterhouses were found. Studies on pigs [23] and poultry [24] indicate that meat inspection data are not readily comparable between countries, and therefore, caution is warranted when comparing international detection rates.

### Dicrocoeliasis

Dicrocoeliasis in the liver was the most frequently detected lesion in our study, with a total prevalence of 2.5% in sheep. Since the findings originated from only eight farms (3.5%), dicrocoeliasis is likely a farm-related problem. A previous study conducted in sheep between 2019 and 2021 in the Iberian Peninsula also suggested that dicrocoeliasis is a farm-related problem [13]. In that study, the prevalence on the most heavily infected farm varied seasonally between 9.1% and 31.2%, whereas on other farms it ranged from 0.0% to 2.7% [13].

Similarly to our findings, *D. dendriticum* has been reported to occur more frequently in organic than conventional sheep in Sweden (5.4% vs. 4.9%) [7] and on the Iberian Peninsula (3.3% vs. 0.0%) [13]. The underlying reasons for the higher prevalence of dicrocoeliasis in organic animals are unclear. Grazing management has been suggested to affect the parasite burden [7]. A lack of organically certified pastures may compel animal keepers to use infected or otherwise high-risk pastures [7, 14]. Varying climatic conditions have also been suggested to affect prevalence [13], whereas wet ground and pastures are unlikely to do so [13, 25].

In our study, dicrocoeliasis was significantly more often detected in adult sheep than in lambs. We found no previous studies comparing the prevalence of dicrocoeliasis between adults and lambs. Unlike fascioliasis, dicrocoeliasis usually does not cause clinical symptoms in sheep and can therefore go unnoticed on farms [26]. Information from the slaughterhouse is thus crucial in identifying infections. Dicrocoeliasis is also often considered only to cause liver condemnations at slaughterhouses and to be of little economic importance; however, more information is needed of its economic impact [26].

### Pleuritis and pneumonia

Pleuritis had the highest farm prevalence (22.4%) in our study, thus being the most widely distributed finding among sheep farms. Pneumonia was found less frequently, with a farm prevalence of 7.0%.

In our study, the prevalence of pleuritis (0.8%) in sheep was higher than the prevalence of pneumonia (0.2%). In previous studies, the detection of pneumonia and pleuritis has varied considerably; in Sweden [7], the prevalences were almost equal (0.7% and 0.8%, respectively), whereas in the UK [8], pneumonia (6%) was more frequent finding than pleuritis (0.5%). Pneumonia can be caused by bacteria from the nasal cavity [27–29], and viruses such as parainfluenza virus 3 and adenoviruses predispose animals to pneumonia [27, 29, 30]. Stress or other predisposing environmental factors are usually required for the onset of pneumonia, which can take from two weeks to several months to develop [27, 31]. Chronic pneumonia rarely shows any symptoms, whereas acute pneumonia typically is associated with clinical signs and can even lead to death within 12 hours [27, 28, 31]. Therefore, most cases of pneumonia observed at the slaughterhouse are probably chronic, as clinically ill animals should not be sent to slaughter. Both acute and chronic pneumonia may lead to pleuritis [27]. Signs of pleuritis, i.e. fibrous adhesions, are permanent, and are thus observable at postmortem inspection [27].

The prevalence of pleuritis, but not pneumonia, was significantly higher in adult sheep than in lambs. Older animals are more likely to have contracted and recovered from pneumonia during their lifetime [8, 9], but the pleural adhesions may remain visible indefinitely [27]. Similarly to a previous Swedish study [7], the prevalence of pleuritis and pneumonia in our study did not differ significantly between organic and conventional production.

### Bone fracture

The majority (97.6%) of the bone fractures found in this study were rib fractures. Rib fractures can result from various types of trauma and may be exacerbated by nutritional imbalances or deficiencies (e.g. Ca/P, vitamin D), which can lead to fragile bones and welfare issues [32–34]. The prevalence of bone fractures did not differ significantly between production types or age groups. Further investigation of nutrition and its effect on rib fractures in Finland is needed.

### Acute bruise

Acute bruises were detected in 36 sheep (0.3%) originating from 15 farms (6.6%). According to the FFA instructions, acute bruises are defined as fresh bruises without yellowish discoloration [15]. Wounds reaching deep into the muscle or joint may also be classified as acute bruises [15]. Such bruises may indicate animal welfare issues related to recent handling of animals at the farm, during transport, or at the slaughterhouse. They can also result from contact with solid structures in the transport vehicle or slaughterhouse.

Based on available recordings, most of the acute bruises in this study were ecchymoses in the abdominal wall (83%). Such bruises or ecchymoses can also be referred to as “blood splashes” which are thought to be caused by elevated blood pressure induced by stressful handling at stunning or delayed onset of bleeding [35]. Stunning itself increases the animal’s blood pressure momentarily and therefore, avoiding additional stress and ensuring a prompt onset of bleeding can decrease the likelihood of blood splashes and partial condemnations [35]. However, it has recently been suggested that also animal husbandry practices on the farm and environmental factors such as annual rainfall affecting the micronutrient content of pasture grass, may also predispose to the sudden rupture of blood vessels during slaughter [36]. Lambs are at a higher risk of ecchymoses than older animals, probably due to their lower stress resilience and more fragile capillaries [36].

Further studies on stunning practices at the slaughterhouses and on nutrition on the farms are needed to determine the causes of ecchymoses. Currently, both ecchymoses likely formed during stunning and bruises caused by trauma can be recorded under the same meat inspection code. Therefore, these different etiologies need to be taken into consideration, if acute bruises are to be used as a welfare indicator on farms.

### Liver abscesses

Liver abscesses were detected in 11 sheep (0.1%) originating from 9 farms (3.9%). Ruminal acidosis caused by carbohydrate-heavy feeding may lead to infection with bacteria and formation of liver abscesses [37–39]. In young animals, omphalitis may also lead to hepatic abscesses [38]. Liver abscesses were detected more often in adult sheep than in lambs and in organic production than in conventional production, but the differences were not significant. We found no previous studies comparing the prevalence of liver abscesses between organic and conventional production or young and old animals.

### Relevance of lesions

The lesions discussed here can be classified further into four main categories: liver lesions (dicrocoeliasis and liver abscesses), pulmonary lesions (pneumonia and pleuritis), lesions possibly related to nutrition (liver abscesses, rib fractures, and ecchymoses in the abdominal wall), and welfare-related lesions (bone fractures and acute bruises). Based on these findings, a possible connection with feeding practices, including use of pastures, should be further studied.

Our results indicate that animal age, as well as management practices and the epidemiological situation on individual farms, affect the meat inspection findings more than production type does. This is in line with earlier studies on differences in animal health between organic and conventional production [12, 40].

### Limitations of the study

Data from a single year and a single slaughterhouse may limit the generalizability of the results. However, using data from a single slaughterhouse reduces variation arising from differences in how meat inspection is performed and findings are recorded across facilities, as shown previously [41–43]. In Finland, sheep are frequently slaughtered in many small slaughterhouses, each differing somewhat in layout, equipment and the personnel performing inspections. Investigating differences between slaughterhouses and their underlying causes would therefore require further studies. Moreover, breed effects were not evaluated in the present study: a previous Finnish study [6] reported only minor differences between breeds, but this matter also warrants further investigation. Nevertheless, the data covered over 20% of the sheep slaughtered annually in Finland, providing a good overview of sheep health and welfare.

Noise caused by the low number of lesions could have been reduced with a larger dataset. However, centrally collected data on all meat inspection results in Finland do not exist. The FFA collects data on whole- and partial-carcass condemnations at the slaughterhouse level, but these data are not linked to, for instance, production type or animal age. Multiple pairwise comparisons were conducted to detect differences between animal prevalences and farm prevalences of different production types and age groups. To handle the rare events and zero values in the data, we used Fisher’s exact test for pairwise comparisons and adjusted the *p*-value for multiple comparisons using the Holm method.

## Conclusions

Based on the low levels of condemnations and recorded meat inspection lesions, sheep reared in Finland can generally be considered healthy, and differences between the animal age groups seem to be greater than those between the production systems. The most common meat inspection lesions can provide information on the health (dicrocoeliasis, pleuritis, pneumonia, and liver abscesses) and welfare (rib fractures and bruises) of animals on farms. Therefore, meat inspection findings may serve as indicators of underlying health and welfare issues at the farm level. However, because nutrition, for instance, can affect the occurrence of rib fractures and liver abscesses, more information on the etiology of these conditions in Finland is needed to elucidate the underlying causes for the detected lesions.

## Data Availability

The datasets used are available from the corresponding author on reasonable request.

## Declarations

### Prior publication

Data have not been published previously.
